# Determination of Thermal, Chemical and Physical Properties of Bedding Materials for Compost Dairy Barns

**DOI:** 10.3390/ani12182450

**Published:** 2022-09-16

**Authors:** Flávio Alves Damasceno, George B. Day, Joseph L. Taraba, Matteo Barbari, Carlos Eduardo Alves Oliveira, Karen Dal Magro Frigeri, Frederico Márcio Corrêa Vieira, Gianluca Bambi

**Affiliations:** 1 Department of Engineering, Federal University of Lavras (UFLA), Lavras 37200-900, MG, Brazil; 2 Department of Agricultural Engineering, University of Kentucky, Lexington, KY 40502, USA; 3 Department of Agriculture, Food, Environment and Forestry, University of Firenze, 50145 Firenze, Italy; 4 Department of Agricultural Engineering, Federal University of Viçosa (UFV), Viçosa 36570-900, MG, Brazil; 5 Biometeorology Study Group, Federal University of Technology—Paraná (UTFPR), Dois Vizinhos 85660-000, PR, Brazil

**Keywords:** chemical properties, composting, dairy cattle, physical properties, thermal properties

## Abstract

**Simple Summary:**

Among animal facilities, compost-bedded pack (CBP) barns have attracted a lot of attention from milk producers and the scientific community. Systematic investigation of the main thermal, chemical, and physical properties of bedding materials in CBP barns is of environmental and economic relevance, helping dairy producers operate these beds properly. Here we assessed 42 CBPs in the state of Kentucky (USA), aiming to study the thermal, chemical, and physical properties of bedding materials. We found that thermal conductivity increased with increasing particle size. Regarding chemical features, the assessed CBPs were similar when considering the bedding materials. The particle weight fraction found in CBPs might result in excessive water retention and low aeration. Based on these main results, we concluded that many dairy producers could use the bedding compost to fertilize their crop fields and avoid over-applying nutrients, and reduce water pollution.

**Abstract:**

The thermal, chemical, and physical properties of compost bedding materials play an important role in every phase of compost production. Based on this, we aimed to assess the thermal, chemical and physical properties of bedding materials for compost-bedded pack (CBP) barns. The database for this study was registered from 42 CBP barns, distributed throughout the state of Kentucky (USA). The thermal conductivity showed a linear relationship with moisture content and bulk density, while thermal resistivity decreased with increasing particle size. The bedding moisture average was 46.8% (±11.5). The average finer index (*p* < 0.05) was the highest weight percentage (30.1%) in the samples studied. Water-holding capacity (WHC) increased with increasingly fine particle size. The higher bulk density value was 3.6 times that of the lowest bulk density value. The chemical characterization of the bedding material provided the following results: 42.7% (±3.8%) C, 1.6% (±0.4%) N, and 28.2 (±8.0) C:N ratio. However, thermal properties are strongly dependent on particle size. Producers can use the bedding material as fertilizer in their crops, due to the chemical characteristics of the materials. Beds with good physical and chemical properties improve their moisture content.

## 1. Introduction

Compost-bedded pack (CBP) barns have been receiving greater attention as alternative housing system for dairy cattle. They consist of a roofed open loose resting area bedded with lignocellulosic material where compost is actively stirred to aerate in order to maintain an active composting process [1]. CBP barn structures generally have a retaining wall that typically surrounds the bed and separates the feed alley from the composted manure pack. The most commonly employed bedding material in the composting process is green and dry sawdust or kiln-dried shavings to maintain a dry bedding surface and to absorb water [2]. However, producers often ask whether green sawdust (high-moisture sawdust from sawing green wood) is harmful or beneficial to the composting bed. The primary difference between green and kiln-dried sawdust is that green sawdust does not absorb as much water as kiln-dried sawdust. Therefore, to achieve the same compost bed moisture content, more green sawdust has to be used than kiln-dried sawdust [2,3].

Therefore, the effectiveness of the composting process is dependent upon the environmental conditions as oxygen content, moisture, temperature, amount of organic matter, and the size and activity of microbial populations present within the CBP [3,4], beyond thermal, chemical and physical properties of the bedding materials [2].

The thermal, chemical and physical properties of compost bedding materials play an important role in every phase of compost production as well as in the handling and utilization of the product. From the mixing of various feedstock, and process monitoring and maintenance, to the packaging and shipping of the final product, parameters such as porosity, bulk density, particle density, moisture content, water-holding capacity and particle size affect the optimum composting environment [5,6] and the design of machinery used in the facility to promote the aeration of the compost bedding material.

Compost thermal, chemical and physical properties are required in many areas of agriculture engineering, agronomy, and animal science. In recent years, considerable effort has gone into developing techniques to determine these properties [7,8,9,10]. Thermal properties of compost bulking materials affect temperature and biodegradation during the composting process. Well-determined thermal properties of compost feedstock will therefore contribute to practical thermodynamic approaches [11,12].

Since a variety of organic materials can be used to make bedding composts (i.e., rice husks, coffee husks, bagasse, paper, peanut shell, wood sawdust and shavings [1,13,14]), knowing their chemical characteristics is very important [15].

Thus, more in-depth study is needed to assess and correlate detailed information regarding thermal, chemical and physical properties of bedding materials and to determine the most significant factors that favor composting success. The findings of this research may be used by current and future CBP managers. The aim of this paper was the evaluation of the thermal, chemical and physical properties of bedding materials for CBP.

## 2. Materials and Methods

### 2.1. Sample Collection

**Figure 1:**
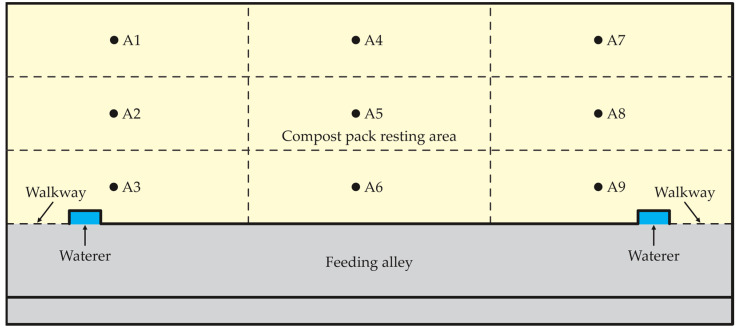
Nine grid spaces (A1 to A9) of sample collections inside the compost-bedded pack barns.

Samples of composting bedding materials were collected from 42 commercial CBP barns distributed throughout the state of Kentucky (USA). In each farm, samples of bedding compost were collected from the surface layer of CBP (0.10 m deep) in nine evenly distributed locations throughout the resting area (Figure 1). Bedding samples were collected using an iron hoe and soil auger. A 20 L container was filled with incremental quantities of bedding collected from the 9 locations to obtain a composite sample of each CBP. The bedding samples were immediately refrigerated upon return to the lab at 1.0 °C. Depending on the type of material used as bedding, the samples collected were classified as: (a) green sawdust, (b) kiln-dried wood shavings or sawdust and (c) mix of both. In this study, 126 samples (42 farms with 3 replicates) of bedding materials were used.

### 2.2. Thermal Properties Measurement

Thermal properties were determined with transient heat dissipation device (KD2, Decagon, Pullman, WA, USA). The thermal properties, thermal conductivity (*k*, W·m^−1^·K^−1^) and thermal resistivity (rho, °C cm·W^−1^), were measured for varying particle size ranges (0.00 mm < Finer < 2.00 mm < PS2 < 4.75 mm < PS3 < 5.60 mm < PS4 < 8.00 mm < Coarser < 25.00 mm) and water content (30, 45 and 60%).

The thermal properties were measured using a handheld device. It consists of a handheld controller and sensors made from thin-wall stainless steel tubing with 2.4 mm diameter × 100 mm long (see KD2 Pro^®^, Operator’s manual, 2008). This probe was inserted into the bedding compost medium.

First, thermal conductivity and thermal resistivity were measured for all samples in different particle size ranges (0.00 mm < Finer < 2.00 mm < PS2 < 4.75 mm < PS3 < 5.60 mm < PS4 < 8.00 mm < Coarser < 25.00 mm), as described earlier.

Wet sample of compost bulking material with different moisture content (30%, 45% and 60%) was produced in a concrete mixer that mixed the bedding material with the added water for 3 minutes. The desired moisture content (30%, 45%, and 60% w.b.) was achieved by adding a known water amount for each particle size range’s initial moisture content, as described by Liberty [16] and Maia et al. [17]. If the initial moisture content (MC) was higher than the selected MC, 50 g of compost was weighed and left to air-dry until the target MC was reached.

### 2.3. Chemical Properties Measurement

Each compost barn pack was subdivided in three equal areas within each of the nine spots (Figure 1) from which 0.4 liters bedding samples were collected during the visit. The bedding samples were collected from the surface and kept cool upon collection at 0 °C until being placed in a —40 °C freezer and held for chemical analyses. Sub-samples were ground as they were, prior to analysis for carbon (C) and nitrogen (N). The analysis was performed at the Division of Regulatory Services at the University of Kentucky. The solid compost was dried in an oven at 75 °C for 24 h, ground to pass a 2 mm screen, and stored at room temperature prior to analysis. Nitrogen (%) and carbon (%) were determined via combustion.

### 2.4. Physical Properties Measurement

Physical analyses including particle size distribution, bulk density, particle density, porosity, water-holding capacity (WHC) were performed to characterize the compost materials.

As-received compost was allowed to air-dry for 48 h before the determination of the particle size distribution. Dried compost was poured in graduated volume cylinders sieved in a sieve shaker (Ro-Tap Model B, W. S. Tyler, Inc., Mentor, OH, USA) with sieves vertically aligned in series in a decreasing mesh screen opening order: 25.00 mm, 8.00 mm, 5.60 mm, 4.75 mm, 2.00 mm and a pan of the bottom. Compost bedding material was sieved in the shaker for 3 minutes. The amount of compost retained by each screen was poured in a beaker and its weight determined.

Compost bulk density was measured by adding 1.6 liters of air-dried compost into a 2.0-liter graduated volume cylinder (0.05 m height and 0.20 m diameter). The container was filled with bedding compost material, and then the material was slightly compacted to ensure absence of large void spaces and, then the material was vibrated using a vibrating Jigsaw (Black & Decker, Model JS515, MD, USA) for 30 seconds. The bulk density can be calculated by dividing the weight of the material by the compost-filled volume of the cylinder [12,17].

Compost particle density was determined by pouring 20 g of as-received compost into five graduated cylinders and adding a portion of 0.10 liter of methanol (90%) to each cylinder up to the 0.10-liter mark. The remaining unused methanol volume represented the volume of particles in the compost-filled cylinder [17].

Porosity was determined for each collected CBP bedding compost from the results of bulk and particle density by using Equation (1):

(1) $$\varepsilon =\left(1-\frac{\rho_{b}}{\rho_{p}}\right)$$

where ε is the overall porosity (in %), $\rho_{b}$ is the bulk density (in g·cm^−3^) and $\rho_{p}$ is the particle density (in g·cm^−3^).

The WHC was determined using 30 grams of the as-received compost that was saturated with deionized water in a beaker, stirred for 3 minutes and left to rest for 7 minutes to absorb water. Saturated compost was poured into three Buchner funnels on top of Erlenmeyer flasks to drain excess water (Whatman #41 filter paper), covered with parafilm, and taken to an environmental chamber at 25 °C and 58% RH, where they drained for 12 h. Drained compost was weighed ($W_{S}$) and dried ($W_{I}$) in a convection oven for 24 h at 105 °C. The water-holding capacity (g water · g dry material^−1^) is calculated as the Equation (2) [12]:

(2) $$WHC=\frac{\left[\left(W_{S}-W_{I}\right)+MC_{I}\cdot W_{I}\right]}{\left[\left(1-MC_{I}\right)\cdot W_{I}\right]}$$

where $MC_{I}$ is the initial moisture content of sample (decimal).

## 3. Results

### 3.1. Thermal Properties of Compost Materials

**Figure 2:**
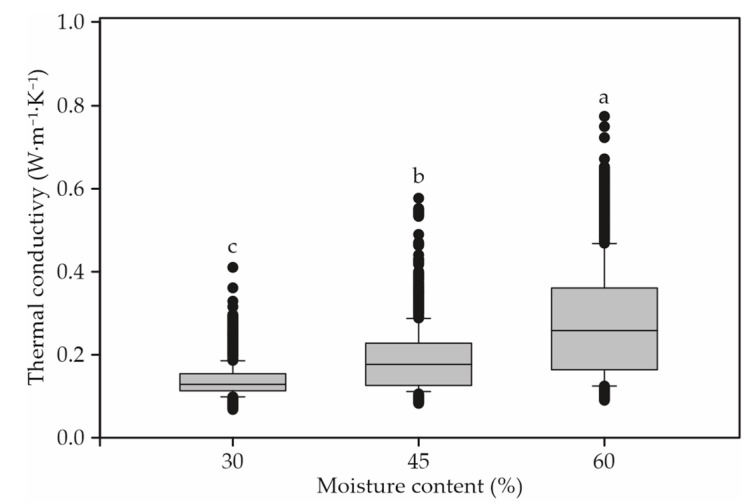
Analysis of variance between thermal conductivity and moisture content for all bed compost materials. Values followed by different letters are significantly different (*p* < 0.05; Tukey).

The thermal conductivity of unsegregated bed compost materials varied from 0.081 to 0.625 W⋅m^−1^⋅K^−1^ for Green sawdust, 0.071 to 0.618 W⋅m^−1^⋅K^−1^ for kiln-dried shavings or sawdust, 0.059 to 0.766 W W⋅m^−1^⋅K^−1^ for mix, and 0.105 to 0.406 W⋅m^−1^⋅K^−1^ for average of all compost material tested, depending upon the moisture content (30, 45, and 60%) within the experimental range of the variables (Figure 2).

An increasing trend in the thermal conductivity of all compost was also observed with the increase in moisture content for all compost material tested (Figure 2).

**Figure 3:**
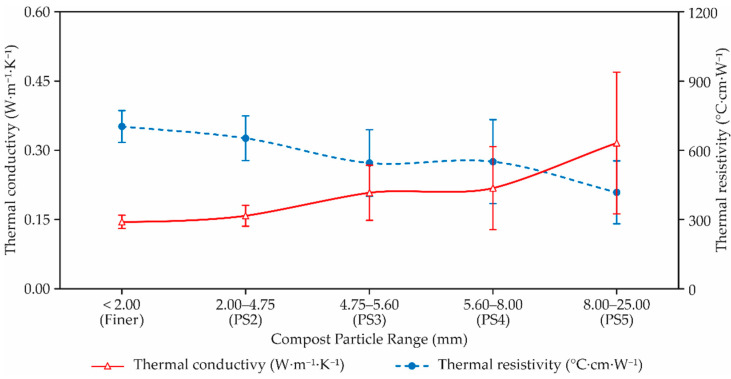
Changes in thermal conductivity (in W⋅m^−1^⋅K^−1^) and thermal resistivity (in °C⋅cm^−1^⋅W^−1^) with different compost particle size of compost materials.

Thermal conductivity and thermal resistivity were plotted versus particle size distribution; these two properties were directly observed data. Values of thermal properties and thermal resistivity were not measured in samples of coarser material because it was not possible to insert the probe of the sensor into the sample. The thermal conductivity increases with the increase of particle size, while thermal resistivity decreases with the increase of particle size. However, the overall thermal conductivity and thermal resistivity varied with each particle size of bed compost material (Figure 3).

### 3.2. Chemical Properties of Compost Materials

**Figure 4:**
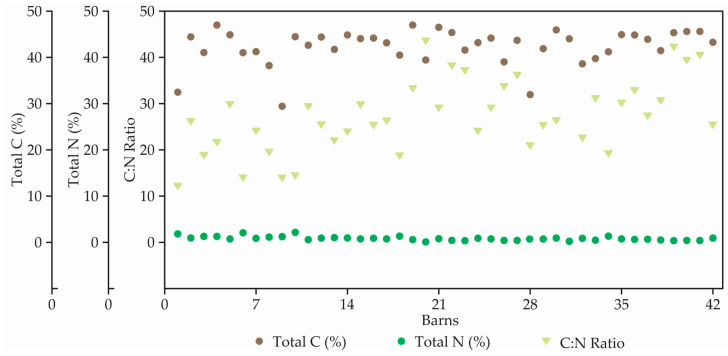
Total Carbon (C, in %), Total Nitrogen (N, in %), and C:N ratio presents in 42 bedding compost.

Results related to total nitrogen (N), total carbon (C), and carbon-nitrogen ratio (C:N) of each bed compost material are shown in Figure 4.

The average total C was 42.7 ± 3.8% with a range of 29.8 to 47.1%. One of the major constituents of the compost, C, was most abundant (47.1%) in CBP barn 19. Only in barn 9, the total average content of C was less than 30.0%. The average total N was 1.6% ranged from 0.5 to 2.5%.

The average C:N ratio was 28.5 ± 8.1. The C:N ratio of barn 10 (15.4) was lowest due to high nitrogen contents as compared to barn 12 (26.2). Barn 20 had the highest C:N ratio (43.9). In general, in the present study the C:N ratio was fairly balanced. However, barns 1, 3, 6, 9, 10, and 18 will require an additional source of nitrogen to obtain the optimum bedding compost C:N ratio between 20 and 25.

### 3.3. Physical Properties of Compost Materials

**Figure 5:**
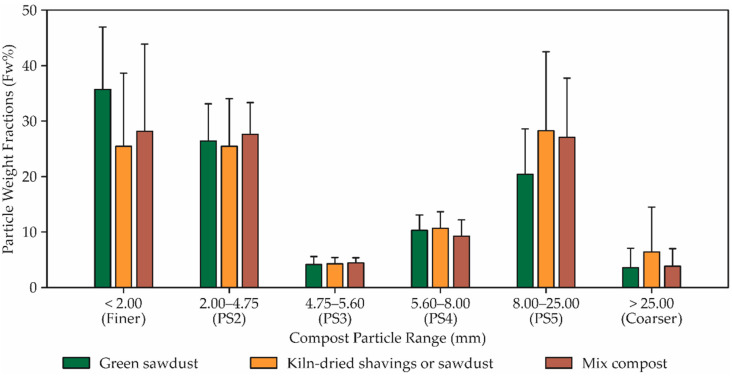
Particle Weight Fractions for compost bedded from CBP barn using Green sawdust, Kiln- dried shavings or sawdust, and Mix compost.

A comparison of the particle weight fraction distributions content (Figure 5) reveals that the values from the CBP barns using different bedding compost materials are very different. The following percentages and standard deviations were found in green sawdust: the particle size ranges (PS2 + PS3 + PS4 + PS5) together made up 60.95 ± 10.0% of the total weight, while coarser material represented 3.46 ± 3.4% and finer material, 35.60 ± 11.3%. In kiln-dried shavings or sawdust, the compost particle size distribution was 25.44 ± 13.1% (finer material), 25.45 ± 8.5% (PS2), 4.12 ± 1.0% (PS3), 10.50 ± 3.0% (PS4), 28.1 ± 14.2% (PS5), and 6.32 ± 8.1% (coarser material). In this plot (Figure 5), the distribution values of mix materials increased in the order of Finer < PS2 < PS5 < PS4 < PS3 < Coarser fractions. In CBP barns using mixed compost material, the lower and higher compost particle size distributions were 14.38% (Coarser > 25.00 mm) and 48.66% (Finer < 2.00 mm), respectively.

**Figure 6:**
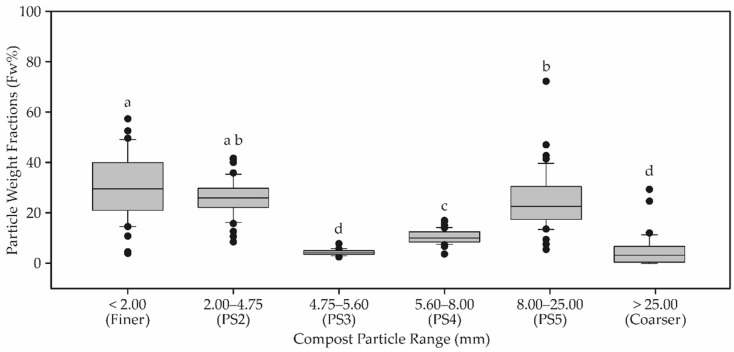
Analysis of variance between compost particle ranges for all bed compost materials. Values followed by different letters are significantly different (*p* < 0.05; Tukey).

The analysis of variance between compost particle ranges from all CBP barns was obtained as shown in Figure 6 at the significance level of *p* < 0.05. The Tukey test shows that there was a significant difference between average values of compost particle ranges. The following percentages and standard deviations were found: in the particle size ranges together (PS2 + PS3 + PS4 + PS5), it comprised 69.5 ± 10.0% of the total weight, while coarser material represented 6.46 ± 3.4% and finer material, 29.60 ± 11.3%. The average finer index expressed as a weight percent in the samples studied was 30.1%.

Particle density, bulk density, and porosity of 42 compost bulking material results are shown in Table 1.

Particle density of all compost material studied ranged from 0.86 to 1.11 g⋅cm^−3^ with an average of 0.98 g·cm^−3^ and a standard deviation of 0.02 (Table 1). Generally, the compost materials of CBP barns 9, 11, 12, 13, 16, 20, 21, 22, 23, 24, 26, 27, 29, 30, 32, 33, 36, 40, 41, and 42 show high particle density (≥g·cm^−3^) compared with other materials (Table 1).

Results and standard errors of bulk density for compost bed material collected from each barn are shown below (Table 1). The average bulk density was 0.38 ± 0.02 g·cm^−3^ with a range of 0.20 to 0.54 g·cm^−3^. Higher bulk density suggests that the compost has less pore space and is more compact. The highest bulk density value was 3.6 times that of the lower bulk density value.

The average porosity of the bedding material in all compost barns was 62.65 ± 2.14% with a range of 47.43 to 77.32%. The variation of average porosity in all compost bulking material was like those bulk density in the reverse order due to the inverse relationship between bulk density and porosity (Table 1).

**Figure 7:**
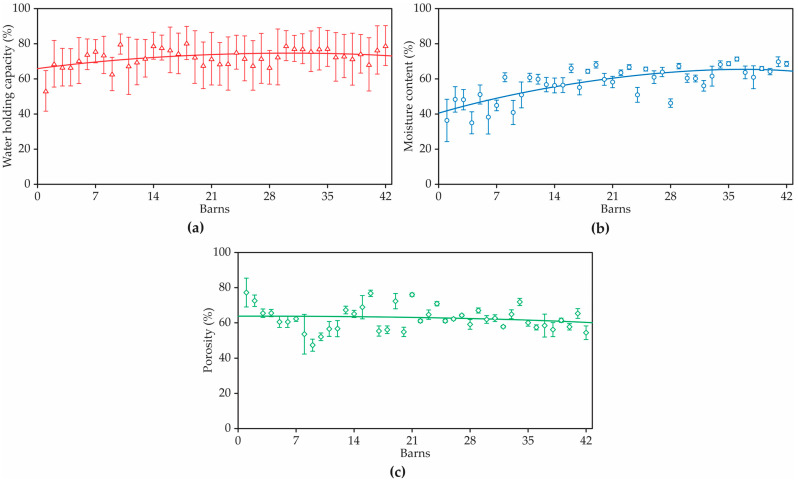
(**a**) Water-holding capacity (in %), (**b**) initial moisture content (in %), and (**c**) porosity (in %) of 42 bed compost materials.

The values of WHC, moisture content and porosity in 42 bed compost materials are illustrated in Figure 7.

The average WHC of all bed compost was at 72.7% on a wet weight basis with a range of 53.6 to 79.8%. WHC was lower for bed compost in CBP barn 1 to the other materials tested and was able to hold water up to 53.6% w.b. moisture content. As expected, the smaller particle size range was able to hold more water (Table 1). WHC increases with decreasing porosity, as can be seen with the joint observation of Table 1 and Figure 7. It was observed that coarse particle sizes of compost have a lower WHC since they are high in large pores and subject to free drainage. There was also a positive relationship between WHC and moisture content (r = 0.383, *p* < 0.05), see Figure 7. Therefore, WHC increased with increasing moisture content.

## 4. Discussion

In this study, we evaluated the thermal (thermal conductivity and thermal resistivity), chemical (C, N and C:N ratio) and physical (particle size distribution, apparent density, particle density, porosity and water retention capacity) of the bed materials used in compost-bedded pack (CBP) barns. Our results showed that the physical properties were more variable than chemical composition, which was similar between the assessed CBPs. The dairy farmers can use the bedding compost to fertilize their crop fields, avoiding over-applying nutrients and reduce water pollution. More details regarding the physical and chemical properties are described below.

### 4.1. Thermal Properties of Compost Materials

Thermal conductivity and thermal resistivity of granular materials are affected by particle contact quality. The number of contacts per particle depends on the packing density, particle shape and particle size distribution. Their effects on thermal conductivity and thermal resistivity are explored in this study using selected compost materials and the thermal needle probe technique.

The thermal conductivity increases with the decrease of particle size [18], while thermal resistivity decreases with the increase of particle size. However, as seen in this study, the overall thermal conductivity and thermal resistivity varied with each particle size of bed compost materials. In general, the thermal conductivity increased with increasing particle size, while thermal resistivity decreased with increasing particle size, which is the exact opposite of what was expected [12,19]. This behavior can probably be explained by considering the higher moisture retention of larger particles once compost materials were air-dried at the same time. It could possibly have changed the values of the thermal properties. Thus, the effect of particle size on thermal conductivity was more pronounced at higher particle size than at lower particle size. However, the trend of thermal conductivity as a function of particle size, in some barns, was very consistent at particle sizes below 4.75 mm. It was also observed that thermal conductivity increased drastically with large particle sizes above 8.00 mm.

The effect of particle size on thermal conductivity was more pronounced for the higher particle sizes than lower particle sizes. However, the tendency of lower thermal conductivity as a function of particle size was very consistent at particle sizes below 4.75 mm. It was also observed that thermal conductivity increased drastically (>85%) with particle sizes larger than 8.00 mm. 

### 4.2. Chemical Properties of Compost Materials

In CBPs it is desirable that there is adequate concentration of C and N in the bed, as micro-organisms require C as an energy source and N as a source of protein [1,20]. In the present study, the mean total C found among CBPs (42.7%) was above that reported by Shane et al. [21] (12.7 a 20.1%). This can be explained because the bed composts in Kentucky have not stabilized to the same extent in this study. In general, many of the beds were not working properly, but as a result, the dairy producers learned how to improve beds to operate correctly.

The total N (11709 kg N) incorporated into the bed throughout the composting period resulted from wood chips (8%) and animal feces (92%) [22]. In the 42 CBPs studied, the mean N value (1.6%) was similar to that reported by other authors, such as 2.54% [23], 0.76% [21], 1.12% [24] and 0.99% [25]. The nitrogen level of compost material is an important factor in the determination of C:N ratio of the composted material. In addition to the C:N ratio, moisture content and pH may affect the bed composting process [1]. Several studies have been conducted in CBP facilities to report the C:N ratio in beds. In Kentucky, USA, Black et al. [20] observed an average ratio of C:N ranging from 16:1 to 35:1. In Brazil, Radavelli et al. [26] found an average ratio of C:N of 10:51. In this context, Shane et al. [21] reported that the highest C:N ratio found was 26.0 during winter. Barberg et al. [27] reported an average of 19.5 and Russelle et al. [24] reported a variation from 11.2 to 20.9. 

The organic matter (OM) of the composting process is degraded more rapidly when the C:N ratio is between 25:1 to 30:1 [28]. However, a C:N ratio below 25:1 may emit ammonia odor [29]. On the other hand, cow feces have a low C:N ratio, ranging from 15:1 to 19:1 [30]. In this way, the use of materials for CBP bed with high C:N ratio becomes extremely necessary. Wood-derived materials have high energy content and a high C:N ratio [1], while barley bark has a low C:N ratio. In this study, the mean C:N ratio found was 28.5. In general, in the present study, it was quite balanced. However, barns 1, 3, 6, 9, 10, and 18 would require an additional nitrogen source to obtain the ideal C:N ratio of the bed compound between 20 and 25. Among the bedding materials that have the highest C:N relationships wood shavings, dry sawdust and fresh sawdust are included [2].

### 4.3. Physical Properties of Compost Materials

Particle size has a direct influence on the growth of micro-organisms [31] and maintaining the porosity of the bed. Particles are classified according to their diameter [32]. In our study, we observed that the particles of the bed materials presented different weight fractions. This variation in distribution values occurred in 10% of the total weight of the green sawdust in particle size ranges between 25.0 to 2.0 mm, 3.4% in the material with greater granulometry (>25.0 mm) and 11.3% of the total weight in the thinnest material (<2.0 mm). In kiln-dried sawdust, the particle size distribution was 13.1% in the thinnest material, 8.5% in the material with 2 mm granulometry, 1.0% in the material with 4.75 mm, 3.0% in the material of 5.60 mm, 14.2% in the material of 8.00 mm and 8.1% in the material with larger granulometry (>25.00 mm). In a prior study, Radavelli et al. [26], when evaluating the particle size in the CBP beds, observed that the material present in the bottom of the sieve represented 29.05%, small material (2 mm) 16.08%, medium material (4.75 mm) 18.36% and large material (9.75 mm) 32.7%. 

Thus, according to the same authors, the size and proportion of particles in the bed has a great influence on the composting process, because in larger particles they have less area and smaller amounts of carbon, limiting the use by micro-organisms. In smaller particles, it increases the likelihood of compaction and availability of oxygen in beds and consequently impairs composting [33]. Thus, further studies are needed to better evaluate and identify the factors that interfere with the properties of bed particles and how management practices can improve these properties. In addition, further studies can help to assist producers in the choice of bedding material and in the economic sustainability of the system.

Variation in particle density between the materials of the beds analyzed was found. In 42% of the sheds, the particles presented higher density compared to other materials. It should be noted that the obtained values are somewhat higher than expected, but evaporation of water increases the solids content, particularly with well-performing beds or high cow density. Cows that spend time in pasture may return soil to the bed. Previous studies report a density of 618.70 kg·m^−3^ [26], 372.7 e 526.2 kg·m^−3^ [14]. However, the daily handling of the bed acts on density and consequently affects bed composting, moisture evaporation and porosity [26]. 

The lower porosity of the bed is the result of materials that absorb a lot of water or urine and, in this way, are not suitable for use, as they limit composting [34]. The average porosity of the material of the beds was 62.65%. The average porosity increased when the bulk density decreased, and significantly higher porosity was found in barn 41 (77.32%).

## 5. Conclusions

The thermal conductivity increased with increasing particle size, while thermal resistivity decreased with increasing particle size. These results indicate that the thermal properties are strongly dependent on particle size, being influenced by the moisture content of the bedding material.

The C:N ratio (28.5) was similar to those reported by other studies that involved bedding materials.

A comparison of the particle weight fraction distributions content reveals that these bedding compost materials found in CBP barns are very different. The distribution of mass weight increased in the order of coarser to finer. The average finer index expressed as a weight percent in the samples studied implied excessive water retention and low aeration. Also, some compost bedding materials showed high particle density compared with other materials.

The porosity is potentially dependent (reverse order) on bulk density although the statistical significance of this relationship was high. Water-Holding Capacity (WHC) increases with increasingly fine particle size.

This investigation showed that compost bedding material with good physical and chemical properties helps to increase the WHC of bedding compost, and can reduce natural drainage and runoff of the moisture (urine and faces) through the surface of the compost, and consequently improves moisture content.

## Figures and Tables

**Table 1:** Particle density, bulk density, and porosity in 42 compost-bulking materials, as well as standard deviations (n = 3).

Barn	Particle Density (g·cm^−3^)	Bulk Density (g·cm^−3^)	POROSITY (%)
1	0.95 ± 0.06	0.50 ± 0.06	77.12 ± 8.23
2	0.98 ± 0.06	0.47 ± 0.01	72.30 ± 3.30
3	0.92 ± 0.04	0.38 ± 0.02	65.41 ± 2.25
4	0.86 ± 0.04	0.31 ± 0.01	65.40 ± 1.84
5	0.93 ± 0.07	0.47 ± 0.03	60.53 ± 3.26
6	0.91 ± 0.05	0.45 ± 0.01	60.50 ± 3.40
7	0.97 ± 0.06	0.37 ± 0.01	62.07 ± 1.65
8	0.99 ± 0.05	0.47 ± 0.13	53.63 ± 11.28
9	1.04 ± 0.05	0.54 ± 0.02	47.43 ± 3.61
10	0.94 ± 0.06	0.45 ± 0.01	51.92 ± 2.30
11	1.01 ± 0.03	0.44 ± 0.04	56.52 ± 4.34
12	1.05 ± 0.07	0.45 ± 0.02	56.70 ± 4.75
13	1.00 ± 0.01	0.35 ± 0.03	67.37 ± 2.18
14	0.99 ± 0.03	0.32 ± 0.01	65.21 ± 2.01
15	0.92 ± 0.03	0.28 ± 0.05	68.64 ± 6.60
16	1.01 ± 0.02	0.24 ± 0.01	76.72 ± 1.57
17	0.98 ± 0.04	0.44 ± 0.01	55.31 ± 2.83
18	0.90 ± 0.02	0.40 ± 0.02	56.00 ± 2.18
19	0.92 ±0.11	0.27 ± 0.01	71.99 ± 4.26
20	1.06 ± 0.01	0.48 ± 0.03	54.81 ± 2.62
21	1.01 ± 0.02	0.24 ± 0.01	75.90 ± 1.02
22	1.00 ± 0.01	0.39 ± 0.01	60.94 ± 0.96
23	1.02 ± 0.06	0.36 ± 0.00	64.73 ± 2.43
24	0.97 ± 0.01	0.28 ± 0.01	70.80 ± 1.10
25	1.00 ± 0.03	0.39 ± 0.01	61.11 ± 0.41
26	1.11 ± 0.01	0.42 ± 0.01	62.29 ± 0.75
27	0.94 ± 0.05	0.34 ± 0.02	64.10 ± 0.94
28	1.04 ± 0.05	0.43 ± 0.01	58.88 ± 2.64
29	1.06 ± 0.04	0.35 ± 0.02	66.83 ± 1.20
30	0.97 ± 0.04	0.37 ± 0.00	61.68 ± 2.11
31	1.04 ± 0.02	0.39 ± 0.01	62.58 ± 1.81
32	1.00 ± 0.00	0.42 ± 0.01	57.80 ± 0.65
33	0.93 ± 0.02	0.33 ± 0.02	64.66 ± 2.52
34	0.97 ± 0.02	0.27 ± 0.02	71.80 ± 2.12
35	1.01 ± 0.01	0.40 ± 0.02	59.73 ± 1.78
36	0.95 ± 0.00	0.41 ± 0.01	57.26 ± 1.44
37	0.91 ± 0.01	0.38 ± 0.06	58.34 ± 6.60
38	0.87 ± 0.04	0.38 ± 0.02	56.11 ± 4.06
39	1.03 ± 0.02	0.40 ± 0.02	61.39 ± 0.78
40	1.06 ± 0.03	0.34 ± 0.03	57.50 ± 1.87
41	1.05 ± 0.00	0.20 ± 0.03	77.32 ± 2.67
42	1.05 ± 0.01	0.32 ± 0.04	54.20 ± 3.78
**Average**	**0.98 ± 0.02**	**0.38 ± 0.02**	**62.65 ± 2.14**

## Data Availability

The data presented in this study are available on request from the corresponding author.
